# A Skill-Based multimodal intervention for dementia caregivers: impact on burden and anxiety

**DOI:** 10.1007/s40520-025-02985-x

**Published:** 2025-03-17

**Authors:** Doaa Said Amin Abdelhalim, Marwa Mostafa Ahmed, Hoda Abdou Hussein, Mai Diaa Sarhan, Ola Osama Khalaf

**Affiliations:** 1 Department of Family Medicine, Faculty of Medicine, Cairo University, Cairo, Egypt; 2 Department of psychiatry, Faculty of Medicine, Cairo University, Cairo, Egypt

**Keywords:** Dementia, Caregiver burden, Anxiety, Psychoeducation, Skill-based intervention

## Abstract

**Background:**

Dementia is a global health challenge affecting both patients and their caregivers, with family members often bearing the primary caregiving burden. In Egypt, where research on caregiver mental health is limited, interventions addressing caregiver burden and anxiety is critical.

**Aim:**

To determine the effect of a psychoeducation and skill-based multimodal intervention on caregiver burden and anxiety.

**Methods:**

A randomized controlled trial (RCT) involving 84 family caregivers. Participants were assigned to either the intervention group (*n* = 42), which received structured psychoeducation and skill-based training over six biweekly sessions, or the control group (*n* = 42), which received routine outpatient care. Caregiver burden and anxiety were assessed using the Zarit Burden Interview (ZBI) and the Generalized Anxiety Disorder Scale (GAD-7). Behavioral and psychological symptoms of dementia were evaluated using the Neuropsychiatric Inventory Questionnaire (NPI-Q).

**Results:**

The sample consisted predominantly of women (79% in the intervention group, 69% in the control group), with daughters forming the largest caregiver subgroup (66.6% and 52.4%, respectively). The mean age of caregivers was 39.2 ± 4.2 years. After three months, caregivers in the intervention group exhibited a significant reduction in burden (ZBI median score: 24 vs. 34, *p* < 0.0001) and anxiety (GAD-7 mean score: 6.15 vs. 9.4, *p* < 0.0001) compared to the control group, however, NPI-Q scores remained unchanged.

**Conclusion:**

A structured psychoeducation and skill-based intervention significantly reduces caregiver burden and anxiety. Implementing such programs in Egypt could improve the well-being of caregivers and individuals with dementia.

**Supplementary Information:**

The online version contains supplementary material available at 10.1007/s40520-025-02985-x.

## Background

The aging global population necessitates addressing neurocognitive disorders like dementia to contain the escalating social and economic costs and improve the well-being of individuals with dementia and their caregivers [1]. The population of individuals with dementia is expected to almost double every 20 years, reaching approximately 152.8 million by 2050, underscoring the urgent need for effective interventions worldwide [2]. This global challenge is acutely felt in Egypt, where dementia prevalence ranges from 1.4 to 21.95%, with projections indicating a dramatic increase [3]. This rapid rise highlights a critical need for research and effective support systems, particularly given the limited resources currently available [4]. Dementia leads to cognitive and functional decline, rendering individuals dependent on caregivers for daily activities. Caregivers also face the immense challenge of managing unpredictable neuropsychiatric symptoms such as agitation, aggression, and disinhibition, which can strain the caregiver-care recipient relationship and exacerbate other caregiving challenges [5]. The combination of managing daily needs, coping with disruptive behaviors, and the lack of improvement in the care recipient’s condition makes this caregiving role exceptionally demanding. Consequently, caregivers face significant levels of burden and emotional stress increasing their risk for developing mental health problems like anxiety [6]. Caregivers of individuals with dementia also, play an essential role in decision-making, and financial management. Studies from Greece have demonstrated that caregiver perceptions significantly influence decisions regarding financial capacity and care, often leading to biases that affect patient outcomes [7].

Similar trends have been noted across Mediterranean cultures, where family-centered caregiving remains the norm. Despite these cultural similarities, Egypt presents unique challenges. Limited public awareness, financial constraints, and gendered caregiving norms place an immense burden on informal caregivers, particularly women [8].

Additionally, caregiver stress has been associated with elder abuse [9], a concern that remains underexplored in Middle Eastern settings.

Studies emphasize the need for a better understanding of dementia, caregiver coping mechanisms, and effective psychological support to alleviate caregiver stress. In Egypt, this need is particularly urgent. The disproportionate burden on female caregivers, stemming from deeply ingrained cultural norms, further compounds the challenges of limited access to specialized dementia care, substantial financial burdens, widespread stigma, and a critical lack of tailored training and support programs [10].

The psychoeducation and skill-based intervention multimodal comprehensive program is a holistic intervention designed to support caregivers of individuals with dementia. It combines education about dementia, communication skills training, and practical caregiving techniques to enhance caregivers’ competence and confidence. By addressing both caregiving challenges and emotional well-being, the program aims to reduce caregiver burden and enhance the care for individuals with dementia [11].

This study aims to assess the impact of this comprehensive program on caregiver burden. The research question guiding this study is: “Does a psychoeducation and skill-based multimodal comprehensive program reduce caregiver burden in those caring for individuals with dementia?” By targeting this question, the research seeks to make a significant contribution to the understanding the effective interventions that support dementia caregivers and enhance the over all health of both dementia caregivers and those living who have dementia.

## Methods

The study utilized a randomized controlled trial (RCT) design, the participants were randomly selected for one of two groups: an intervention group (who were given a psychoeducation and skill-based multimodal comprehensive program) or a control group (who were given standard care), ensuring equal group sizes.

### Settings, sampling, and participants

The research was carried out at geriatric outpatient clinic of a psychiatric hospital in Cairo University. Data collection occurred between May 2023 and January 2024. The attending psychiatrist diagnosed dementia based on DSM-5.The study’s target population consisted of adult family caregivers tasked with providing home care for individuals diagnosed with mild to moderate dementia based on DSM-5 criteria. Only those who fulfilled certain inclusion criteria were recruited.

Inclusion criteria.

- They were responsible for providing home care for individuals who had dementia.
- They were 19 year of age(aligning with legal adulthood) or older.

The study excluded caregivers if:

- They were caring for individuals who had severe dementia according to DSM-5.
- They were caring for individuals who had another severe psychiatric conditions.
- Being paid for their caregiving role.
- Caregivers were unwilling to attend follow-up sessions.
- Caregivers were experiencing moderate to severe depression.
- Caregivers were undergoing psychiatric treatment.

To ensure statistically significant results, the study’s minimum sample size was determined using assumptions derived from a previous study in this field.(Kobayashi & Honda, 2021) [10] using Epi-calc 2000 software. Assuming 80% power, a 0.05 level of significance, a mean burden score of 13.3 before intervention, a mean burden score of 10.6 after intervention, and a range of 3, the minimum sample size was 38 participants. To compensate for an estimated 10% dropout rate, the researchers aimed for a final sample size of 42 participants per group.

### Measures

The study utilized the Arabic versions of the Zarit Burden Interview (ZBI), the Generalized Anxiety Disorder Scale (GAD−7), and the Neuropsychiatric Inventory Questionnaire (NPI-Q).

1. Arabic Zarit Burden Interview (ZBI): This tool measures the caregiver’s burden while providing care for an individual with dementia, focusing on emotional well-being, physical health, social life, and financial impact. Originally developed by Zarit et al., it has been widely translated and validated across various cultures. Scores range from 0 to 88, with higher scores indicating a greater level of caregiver burden [11–13].Cronbach’s α = 0.89 (current sample).
2. Arabic Generalized Anxiety Disorder Scale (GAD−7): A self-administered instrument used to assess anxiety symptoms, the GAD−7 contains seven items evaluating the frequency and severity of anxiety over the two weeks prior to the survey. On this anxiety scale, individual items are scored 0–3, resulting in a total score between 0 and 21. A score of 10 or above indicates clinically significant anxiety [14]. Cronbach’s α = 0.86.
3. The Arabic Neuropsychiatric Inventory Questionnaire (NPI-Q) is a caregiver-reported assessment tool, developed and validated by Feghali et al. (2021). It screens for 12 neuropsychiatric symptoms in people with dementia, such as delusions, hallucinations, and anxiety. For each symptom, the caregiver notes whether it occurred in the last month and rates its severity from mild to severe. The resulting score reflects the overall symptom burden, with a potential range of 0 to 36 [15, 16]. Cronbach’s α = 0.84.

### Data collection

Data was collected through paper-based Arabic questionnaires administered at the geriatric clinic during scheduled appointments. Researchers facilitated the process by providing instructions and ensuring participants comprehended the questions. All responses were anonymized using unique identification numbers, ensuring the removal of personal information. The research team maintained secure handling and storage of the data.

### Study procedures

**Fig. 1:**
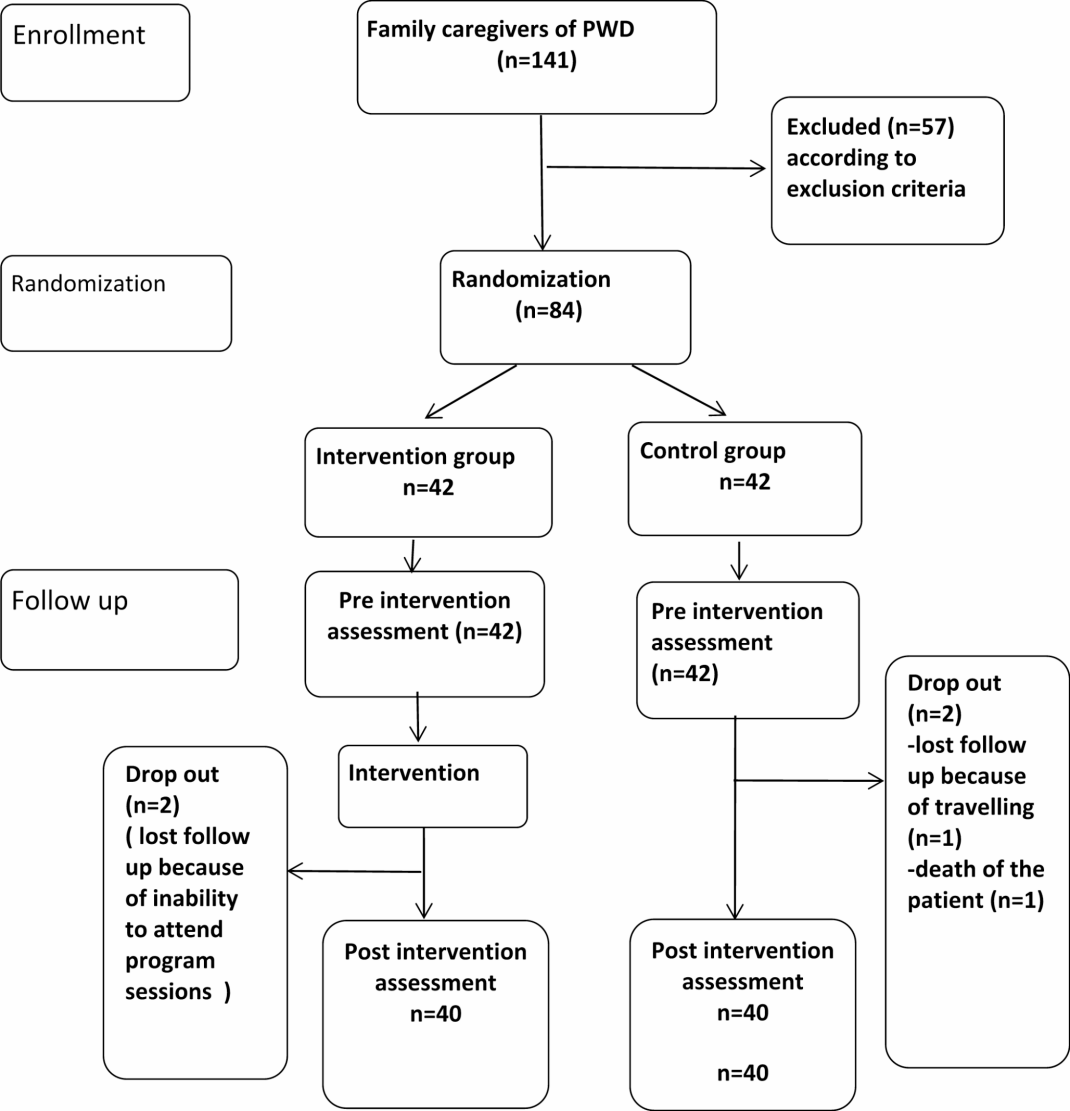
Flow chart of the study participants

Researchers contacted family caregivers who agreed to participate via telephone. Participants who met the inclusion and exclusion criteria were randomly assigned to one of two groups using a computer-generated randomization process participants were allocated as shown in Fig. 1.

### Intervention program

The program was designed to empower caregivers of people with dementia. This comprehensive program provided caregivers with the tools and training required to succeed in their responsibilities. Delivered over six biweekly sessions, each lasting 1.5 to 2 h, using a multifaceted approach that included: Face-to-face health education, Educational videos, Printed materials.

The sessions covered the following topics:

Session 1: Brainstorming personal experiences, education on dementia and its impact on caregivers.

Session 2: Communication skills, common communication difficulties, and managing behavioral problems.

Session 3: Basic information about dementia and Alzheimer’s disease, and caregiving tips.

Session 4: Common dementia behaviors and strategies for management.

Session 5: Integrating communication skills into daily caregiving, guidance on healthy nutrition, dressing, and bathing.

Session 6: Integrating communication skills, realistic expectations, self-care, and transferring acquired knowledge and skills.

The intervention program’s comprehensive approach aimed to empower caregivers and Improve their understanding of dementia and how to best meet the needs of their loved ones living with the condition. Further details on the intervention protocol, including session content and materials, are provided in the Supplementary File.

Control Group: The control group received standard dementia care, which consisted of routine medical follow-ups at the geriatric outpatient clinic, including basic information about dementia, caregiving, safety, and community resources. They received monthly follow-up visits to address their needs. Caregivers in this group were provided with general health advice regarding dementia management and access to informational pamphlets about the condition. However, they did not receive structured psychoeducation, skill-based training, or interactive support sessions.

.

### Statistical analysis

Data were analyzed using SPSS (version 21). Descriptive statistics included frequencies, percentages, means, and standard deviations. The chi-square test was applied to compare categorical variables, and the Fisher exact test was used when expected cell frequencies were less than five. For quantitative variables, a one-sample t-test was used if the data followed a normal distribution, while the Mann-Whitney U test was used for non-normally distributed data. A p-value of ≤ 0.05 was considered statistically significant.

## Results

The study involved 84 adult family caregivers for individuals with mild to moderate dementia. The intervention and control groups were comparable in terms of key demographic and social characteristics.

The median age in the intervention group was 39 years (IQR 34.5–42), and in the control group, it was 40 years (IQR 36.5–43). Most participants were female (79% in the intervention group and 69% in the control group). Regarding education, the majority in both groups had a secondary education (60% in the intervention group and 52% in the control group), while a smaller proportion had a tertiary education (14% in both groups) (Table 1).

Regarding the demographics of the dementia patients, there was no statistically significant difference in the age, gender distribution, previous occupation and marital status between the intervention and control groups. The two groups were matched regarding the age and sex (p value > 0.05) as median age of the intervention group was 71.5 (68–73) years and that of controls was 71.5 (68–74) with the majority of both groups were females. The majority of both groups were married, 66.7% for the intervention group and 61.9% for the control group. The average number of years since diagnosis was around 2.5 years in both groups and the majority of patients had moderate disease (Table 2).

At baseline, both groups had similar levels of emotional and psychological distress. Most participants reported mild to moderate caregiver burden and experienced anxiety. No statistically significant differences were observed between the groups in perceived burden (Zarit Burden Interview), anxiety levels (GAD-7), or neuropsychiatric symptom severity (NPI-Q) at baseline (Table 3).

After a three-month intervention, the intervention group showed a significant reduction in caregiver burden (Zarit Burden Interview) (*P* < 0.0001), anxiety levels (GAD-7) (*P* < 0.0001), and neuropsychiatric symptom severity (NPI-Q) (*P* < 0.05). In contrast, no significant changes were observed in the control group regarding caregiver burden, anxiety, or neuropsychiatric symptom severity before and after the intervention (Table 4).

When comparing post-intervention outcomes between the two groups, the intervention group demonstrated a significantly greater reduction in caregiver burden (Zarit Burden Interview) (*P* < 0.0001) and anxiety levels (GAD-7) (*P* < 0.0001) than the control group. However, there was no significant difference in neuropsychiatric symptom severity (NPI-Q) between the groups after the intervention **.**Reduction in caregiver burden (ZBI): Cohen’s d = 1.84 (large effect). Reduction in anxiety (GAD-7): Cohen’s d = 1.71 (large effect) (Table 5).

Female caregivers showed a significant reduction in burden and anxiety after the intervention (*p* < 0.001), whereas male caregivers did not experience a statistically significant change. Higher-educated caregivers benefited more from the intervention (*p* < 0.001) compared to those with only basic education (*p* = 0.02) (Table 6).

**Table 1:** Baseline characteristics of the study population(Caregivers) of both groups

	Intervention group (*n* = 42)	Control group (*n* = 42)	*P*
**Age** Median (IQR)	39.2 (34.5–42)	40.5 (36.5–43)	0.2(NS)
**Sex** Male n (%) /Female n (%)	9 (21.4%) /33 (78.6%)	13 (31%)/ 29(69%)	0.2(NS)
**Relationship with the patient**			
Son n (%)	5 (12%)	13 (31%)	0.1(NS)
Daughter n (%)	28 (66.6%)	22 (52.4%)	
Wife n (%)	4 (9.5%)	6 (14.2%)	
Husband n (%)	0	0	
Sister n (%)	2 (4.8%)	1 (2.4%)	
Brother n (%)	3 (7.1%)	0	
**Education**			
Illiterate n (%)	0	2 (4.8%)	0.7(NS)
Basic education n (%)	11 (26.3%)	12 (28.6%)	
Secondary n (%)	25 (59.5%)	22 (52.4%)	
Faculty/Institute n (%)	6 (14.2%)	6 (14.2%)	
**Occupation**			
Non-working n (%)	29 (69%)	25 (59.5%)	0.2(NS)
Manual worker n (%)	5 (12%)	11 (26.3%)	
Professional n (%)	8 (19%)	6 (14.2%)	
**Marital status**			
Single n (%)	3 (7.1%)	2 (4.8%)	1(NS)
Married n (%)	39 (92.9%)	40 (95.2%)	

**Table 2:** Demographics and clinical characteristics of dementia patients in the two study groups

		Intervention group (*n* = 42)		Control group (*n* = 42)	*P*
**Age** Median (IQR)		71.5 (68–73)		71.5 (68–74)	0.4(NS)
Gender Male n (%) /Female n (%)		15 (35.8%) / 27 (64.2%)		12 (27.6%)/ 30 (71.4%)	0.5(NS)
**Previous occupation**					
Non-working n (%)		26 (61.9%)		31 (73.8%)	0.1(NS)
Manual worker n (%)		13 (31%)		6 (14.2%)	
Professional n (%)		3 (7.1%)		5 (12%)	
**Marital status**					
Single n (%)		4 (9.5%)		4 (9.5%)	0.9(NS)
Married n (%)		28 (66.7%)		26 (61.9%)	
Widowed n (%)		10 (23.8%)		12 (28.6%)	
**years since diagnosis** Mean (SD)	2.75 (1.30)		2.6 (1.13)		0.6(NS)
**Type of Disease**					
Mild n (%)	13 (30%)		16 (40%)		0.3(NS)
Moderate n (%)	29 (70%)		26 (60%)		

**Table 3:** Baseline burden, anxiety and NPI Q severity of the two studied groups

	Intervention group (*n* = 42)	Control group (*n* = 42)	*P*
**Zarit Burden Interview**	35 (28–42)	33 (20−41.5)	0.5(NS)
**0–20** no burden	8	13	0.5(NS)
**21–40** mild to moderate	22	18	
**41–60** moderate to severe	11	11	
**61–88** Extreme burden	1	0	
**NPIQ severity** Median (IQR)	15 (8–18)	14 (7.5–18)	0.1(NS)
**GAD−7** Mean (SD)	9.78 (2.92)	9.71(2.13)	0.3(NS)
Minimal (0–4)	-	-	0.4(NS)
Mild (5–9)	26	24	
Moderate (10–14)	16	18	

**Table 4:** Pre & post-intervention comparison of caregiver’s burden, anxiety and NPI Q severity among the intervention and the control groups

	Intervention group (*n* = 40)			Control group (*n* = 40)		
	Pre	Post	P	Pre	Post	P
**Zarit burden** Median (IQR)	35 (28–42)	24 (19-26.5)	**< 0.0001**	33 (20-41.5)	34 (26.5–38)	0.4
**GAD-7** Mean (SD)	8.78 (2.62)	6.15 (2.13)	**< 0.0001**	9.38 (2.14)	9.4 (1.81)	0.9
**NPIQ severity** Median (IQR)	15.5 (8.5–19)	15 (8–18)	**0.03**	14 (7–17)	14 (7.5–18)	0.1

**Table 5:** Comparison betweeen the change in caregivers’ burden, anxiety and depression indicators in the intervention and the control groups after intervention

	Follow-up scores in intervention group	Follow up scores in control group	*P* value	Effect Size (Cohen’s d)
**Zarit burden** Median (IQR)	24 (19-26.5)	34 (26.5–38)	**< 0.0001**	**1.84**
**GAD-7** Mean (SD)	6.15 (2.13)	9.4 (1.81)	**< 0.0001**	**1.71**
**NPIQ severity** Median (IQR)	15 (8–18)	14 (7.5–18)	0.8	

**Table 6:** The relation between demographic variables and caregiver burden and anxiety

Variable	Category	ZBI: Intervention (Mean ± SD)	ZBI: Control (Mean ± SD)	*P*-value	GAD-7: Intervention (Mean ± SD)	GAD-7: Control (Mean ± SD)	*P*-value
Gender	Male	32.1 ± 5.8	34.7 ± 6.2	0.4 (NS)	8.1 ± 2.4	9.2 ± 2.1	0.3 (NS)
	Female	24.8 ± 4.3	33.5 ± 5.1	**< 0.001**	5.2 ± 1.9	8.8 ± 2.3	**< 0.001**
Education Level	Basic	27.5 ± 5.2	35.2 ± 5.7	0.02	7.0 ± 2.3	9.1 ± 2.6	0.04
	Higher	23.1 ± 3.9	32.8 ± 4.6	**< 0.001**	4.9 ± 1.7	8.6 ± 2.2	**< 0.001**
Relationship to Patient	Daughter	25.3 ± 4.5	33.7 ± 5.4	**< 0.001**	5.5 ± 2.0	8.7 ± 2.5	**< 0.001**
	Son	30.2 ± 5.1	34.5 ± 5.8	0.3 (NS)	7.8 ± 2.2	9.0 ± 2.4	0.35 (NS)

## Discussion

Dementia imposes a substantial burden on individuals, families, the healthcare system, and the economy. It leads to a progressive decline in cognitive and functional abilities, requiring individuals with dementia to depend heavily on caregivers for daily tasks [17]. This dependency, coupled with challenging behaviors, necessitates constant supervision, placing significant stress on caregivers [18].

In Egypt, where strong family ties are highly valued, caregiving often falls to family members. This cultural expectation amplifies the burden, particularly for women, who bear the brunt of caregiving responsibilities due to societal norms and traditional gender roles [19]. Caregiver support is essential from the early stages of dementia, and the demands grow as the disease progresses, persisting until the end of life [20]. Although relatively new in Egypt, multimodal approaches show promise by providing caregivers with essential knowledge and skills, enhancing their caregiving capabilities, and alleviating caregiver stress [21].

Our study demonstrated a statistically significant improvement (p-value < 0.05) in the Zarit Burden Interview (ZBI) scores for the intervention group compared to the control group after the intervention. The ZBI median (IQR) scores were 34 (26.5–38) and 24 (19–26.5) for the control and intervention groups, respectively, indicating the program’s effectiveness. This aligns with findings from Cheung et al. (2015) [22], who reported reduced subjective burden following a multicomponent intervention, and Arango-Lasprilla et al. [23], who observed lower burden scores in the treatment group during post-tests and three-month follow-ups compared to the control group.

In our study, caregivers received extensive training on dementia care, including practical strategies tailored to the unique challenges faced by Egyptian families. These sessions emphasized culturally sensitive communication techniques and coping mechanisms. Group training further provided caregivers with an opportunity to connect with others in similar situations, fostering a sense of community and reducing feelings of guilt and isolation—common issues within Egypt’s collective family structure.

A statistically significant difference (p-value < 0.05) was also observed in GAD-7 scores between the intervention and control groups at follow-up. The mean (SD) scores were 9.4 (1.81) and 6.15 (2.13) for the control and intervention groups, respectively. Previous studies have highlighted the mental health benefits of caregiver interventions, including group discussions and educational support, in reducing anxiety and burden [24]. Walter et al.’s meta-analysis [25] similarly concluded that such programs improve caregiver anxiety. Given the limited access to mental health resources in Egypt, our findings emphasize the importance of expanding such programs nationwide. However, Tamura et al. [26] did not find significant improvements in caregiver anxiety, underscoring the variability in outcomes across different settings.

For NPI-Q severity scores, no significant difference was noted between the intervention and control groups at follow-up, with median (IQR) scores of 14 (7.5–18) and 15 [8–18], respectively. However, significant improvements were observed within the intervention group pre- and post-intervention (p-value < 0.05) for ZBI, NPI-Q, and GAD-7 scores, while no such changes occurred in the control group.

The intervention group showed a marked reduction in ZBI scores after the program, with median (IQR) scores decreasing from 35 (28–42) to 24 (19–26.5). GAD-7 scores also significantly improved, with mean (SD) scores dropping from 8.78 (2.62) to 6.15 (2.13), demonstrating the program’s effectiveness in reducing both caregiver burden and anxiety.

Baseline NPI-Q severity scores were 15 [8–18] and 14 (7.5–18) for the intervention and control groups, respectively. Approximately 90% of individuals with dementia experience at least one behavioral or psychological symptom [27]. This finding highlights the need for caregiver interventions addressing these symptoms, especially in Egypt, where limited mental health resources often leave families without adequate support.

The majority of caregivers in the study were women, reflecting cultural norms in Egypt that associate caregiving with women. Additionally, women are more likely to access healthcare services and attend follow-up visits, further contributing to this gender disparity [28, 29].

Our findings offer valuable insights for public health and clinical practice in Egypt. They suggest that a psychoeducation and skill-based multimodal programs have significant potential to improve the well-being of caregivers and individuals with dementia. However, scaling these interventions will require increased awareness, culturally tailored training materials, and government support to ensure their availability across urban and rural regions.

### Study strength

This study employed a rigorous randomized controlled trial (RCT) design, which helps establish causality and minimize bias. The inclusion of a control group receiving standard care allowed for a direct comparison to the intervention group, strengthening the study’s internal validity. Standardized measures, like the Zarit Burden Interview, were used to ensure reliability and objectivity in assessing caregiver burden. The study’s findings, demonstrating a significant reduction in caregiver burden after the intervention, suggest the effectiveness of the psychoeducation and skill-based multimodal comprehensive program and its potential to improve the well-being of both caregivers and individuals with dementia. This research contributes to the growing body of knowledge on interventions for dementia care and informs public health strategies for supporting caregivers.

## Limitations

The study only assessed outcomes immediately after the intervention, leaving the long-term impact unclear. Future research should focus on longitudinal follow-up to assess sustained benefits, as well as adaptation of the program for rural communities.This study is limited by its single-site recruitment, which may not fully represent Egypt’s caregiver population.

Additionally, blinding was not feasible in a psychoeducational intervention, potentially introducing assessment bias. Future research should address these limitations by conducting multi-site studies and incorporating objective stress measures (e.g., cortisol levels).

## Conclusion

The study demonstrated the effectiveness of the psychoeducational and skill based multimodal comprehensive program in reducing caregivers’ perceived burden. Significant improvements were observed in caregivers’ burden scores after the intervention compared to the control group. Caregivers also experienced significant improvements in anxiety scores after the intervention. The neoropsychiatric symptoms of dementia showed no change after the intervention compared to the control group. These findings underscore the importance of targeted interventions for caregivers to mitigate the negative consequences of dementia care. Implementing caregiver-focused interventions like this into routine care will enhance the overall quality of care for individuals with dementia and their families.

## Recommendations

Future research should prioritize longitudinal studies to understand how caregiver burden and anxiety evolve over time. Additionally, there’s a critical need to develop culturally tailored interventions that address specific challenges, provide education and skills training, and promote caregiver well-being and resilience. This requires comprehensive training for healthcare professionals and social workers on the importance of caregiver support and evidence-based intervention strategies. Developing educational materials and resources for families is essential to equip them with the knowledge and skills necessary for managing dementia care effectively. Furthermore, policymakers must prioritize resource allocation to enhance support for caregivers of individuals with dementia. This includes establishing comprehensive support systems with respite care, financial assistance, and access to evidence-based interventions. By addressing these areas, we can create a more supportive and effective system of care for both individuals with dementia and their caregivers.

## Electronic supplementary material

Below is the link to the electronic supplementary material.

Supplementary Material 1

## Data Availability

No datasets were generated or analysed during the current study.
