# Supplementary material for: A Skill-Based multimodal intervention for dementia caregivers: impact on burden and anxiety
Source: Aging Clin Exp Res. 2025 Mar 17;37(1):95. doi: 10.1007/s40520-025-02985-x (PMC11914238; doi:10.1007/s40520-025-02985-x)
Supplement: Supplementary file 1 — Supplementary Material 1 [file 40520_2025_2985_MOESM1_ESM.docx]

# **Detailed Description of the Intervention Program**

## **1. Overview of the Intervention**

This intervention was designed as a structured psychoeducation and skill-based support program for family caregivers of dementia patients. The primary aim was to reduce caregiver burden and anxiety while improving caregiving competencies. The intervention consisted of six biweekly sessions, each lasting approximately 1.5 to 2 hours, delivered in small-group settings (6–8 caregivers per group). Sessions were conducted by trained dementia care specialists, including geriatric psychiatrists and clinical psychologists.

## 2. Session-by-Session Breakdown

### **Session 1: Understanding Dementia and Caregiver Burden**

Overview of dementia types, disease progression, and cognitive symptoms. Caregivers were educated on the emotional, physical, and financial burdens of caregiving. Strategies for managing stress and expectations were introduced.

### Session 2: Communication Strategies and Behavior Management

Training on effective communication with dementia patients, including verbal and non-verbal techniques. Techniques for handling agitation, aggression, and repetitive questioning were practiced through role-playing exercises.

### Session 3: Practical Caregiving Techniques

Guidance on activities of daily living (ADLs), such as dressing, feeding, and toileting assistance. Demonstration of mobility aids and strategies for maintaining patient independence.

### Session 4: Addressing Neuropsychiatric Symptoms

Education on common neuropsychiatric symptoms, including depression, hallucinations, and sleep disturbances. Strategies for de-escalating distress and reducing caregiver burnout were discussed.

### **Session 5: Nutrition, Hygiene, and Physical Care**

Best practices for ensuring proper nutrition and hydration in dementia patients. Training on hygiene routines and safe physical care, including fall prevention techniques.

### **Session 6: Integrating communication skills, realistic expectations, self-care, and transferring acquired knowledge and skills**

Encouragement to seek support from community and professional services. Training on stress management techniques and the importance of self-care. Final Q&A and personalized caregiving strategies were provided.

## 3. Materials Used in the Intervention

### Printed Handouts

Caregiving guides covering dementia progression, behavior management, and self-care techniques.

### Educational Videos

Demonstrations of caregiving techniques, including safe patient handling and communication strategies.

### Interactive Role-Playing

Practical exercises for caregivers to practice communication and behavioral management skills.

### Visual Aids

Step-by-step instructions on ADL assistance and home safety modifications.

### Caregiver Workbooks

Session-based worksheets for self-assessment

### Support Group Discussions

Facilitated discussions to share experiences, coping mechanisms, and problem-solving approaches.

## 4. Implementation Strategy

Caregivers attended in-person sessions at a hospital-affiliated dementia care unit.
Each session followed a structured format: (1) A 20-minute educational lecture, (2) A 40-minute interactive discussion, (3) A 30-minute skill-building exercise. Participants were provided with supplementary materials for home use.

## 5. Reproducibility and Future Applications

This intervention model can be adapted for different cultural and healthcare settings.
Key recommendations for replication include ensuring trained facilitators, providing culturally relevant materials, and integrating community support services.
